# Circulating exosomes deliver free fatty acids from the bloodstream to cardiac cells: Possible role of CD36

**DOI:** 10.1371/journal.pone.0217546

**Published:** 2019-05-29

**Authors:** N. A. Garcia, H. González-King, E. Grueso, R. Sánchez, A. Martinez-Romero, B. Jávega, J. E. O’Connor, P. J. Simons, A. Handberg, P. Sepúlveda

**Affiliations:** 1 GECORP, Buenos Aires, Argentina; 2 Regenerative Medicine and Heart Transplantation Unit, Instituto de Investigación Sanitaria La Fe, Valencia, Spain; 3 Joint Research Unit for Cardiovascular Repair IISLAFE-CIPF, Valencia, Spain; 4 Joint Research Unit of Cytomics CIPF-UVEG, Valencia, Spain; 5 Department of Biochemistry, University of Valencia, Valencia, Spain; 6 Bioceros BV, Utrecht, The Netherlands; 7 Department of Clinical Biochemistry, Aalborg University Hospital, Aalborg, Denmark; 8 Department of Clinical Medicine, Faculty of Health, Aalborg University, Aalborg, Denmark; University of Alabama at Birmingham, UNITED STATES

## Abstract

Regulation of circulating free fatty acid (FFA) levels and delivery is crucial to maintain tissue homeostasis. Exosomes are nanomembranous vesicles that are released from diverse cell types and mediate intercellular communication by delivering bioactive molecules. Here, we sought to investigate the uptake of FFAs by circulating exosomes, the delivery of FFA-loaded exosomes to cardiac cells and the possible role of the FFA transporter CD36 in these processes. Circulating exosomes were purified from the serum of healthy donors after an overnight fast (F) or 20 minutes after a high caloric breakfast (postprandial, PP). Western blotting, Immunogold Electron Microscopy and FACS analysis of circulating exosomes showed that CD36 was expressed under both states, but was higher in postprandial-derived exosomes. Flow cytometry analysis showed that circulating exosomes were able to take-up FFA directly from serum. Importantly, preincubation of exosomes with a blocking CD36 antibody significantly impeded uptake of the FFA analogue BODIPY, pointing to the role of CD36 in FFA exosomal uptake. Finally, we found that circulating exosomes could delivery FFA analogue BODIPY into cardiac cells *ex vivo* and *in vivo* in a mice model. Overall, our results suggest a novel mechanism in which circulating exosomes can delivery FFAs from the bloodstream to cardiac tissue. Further studies will be necessary to understand this mechanism and, in particular, its potential involvement in metabolic pathologies such as obesity, diabetes and atherosclerosis.

## 1. Introduction

Circulating free fatty acids (FFAs) are thought to be the major source of lipid fuel in the body, and are crucial to the energy metabolism of the renal cortex [1], myocardium [2], liver [3], and resting skeletal muscle [4]. It is well recognized that circulating FFAs are almost exclusively derived from the adipose tissue (AT) through the hydrolysis of triglycerides (TGs) [5]. Endothelial lipases, principally lipoprotein lipase (LPL), also contribute to the circulating FFA pool, especially after ingestion of a fat meal, by hydrolysing circulating TGs carried in chylomicrons in the capillaries of AT. For the most part, released FFAs are taken-up by adipocytes for storage, but a proportion generally escapes and contributes to the circulating FFA pool [6] in a process called spillover. This process may constitute 40–50% of the total circulating FFAs in the postprandial period [7].

Exosomes are nanovesicles (20–150 nm) of endocytic origin that are secreted into circulation by a wide variety of cells and have roles in paracrine signalling both in normal physiological conditions [8], and also in pathological conditions such as tumour progression [9]. Exosomes transport a range of molecules including DNA, RNA, proteins, hormones and lipids, and they are also involved in the transcellular transport of phospholipases and prostaglandins [10]. Moreover, exosomes have been found in circulation, and likely play important roles in physiopathological processes [11].

Circulating exosomal uptake of FFAs has not previously been investigated. Here we show that circulating exosomes present in blood from healthy donors express the fatty acid transporter CD36. CD36 is also known as FAT (fatty acid translocase) because it binds long chain free fatty acids and facilitates their transport into cells [12]. In adipocytes CD36 contributes to lipid storage. In cardiac cells, it serves to supply the cells of fuel for beta-oxidation, the main source of energy for heart contraction. We observed that CD36 levels significantly increase in exosomes harvested in the postprandial (PP) as compared with the fasting state (F), resulting in an increase in the uptake of FFAs and also in their subsequent delivery to cardiac cells. Overall, we provide evidence supporting a new exosome-mediated mechanism of circulating FFA delivery from blood to tissues.

## 2. Materials and methods

The ethics committee of the IISLaFe, Valencia, Spain, reviewed and approved the study that we are presenting. Approval number: 2016/0763. Form of obtained: oral.

### 2.1 Animals

Nod/scid mice and Wistar rats were purchased from Charles River Laboratories Inc. (Wilmington, MA). The management of the animals was carried out according to the requirements stipulated by the Royal Decree 53/2013 on the protection of animals used for experimentation and other scientific purposes, and in compliance with all the rules and recommendations of the Ethics and Animal Welfare Committee (CEBA) of the Hospital La Fe Research Fundation and Príncipe Felipe Research Institute. Wistar rats were used as breeders, and pups were used for isolation of rat primary cardiomyocytes (CM). Nod/scid mice were used for the *in vivo* experiment described in section 2.12.

### 2.2 Study design

Healthy male and female donors (20–40 years of age) with a body mass index (BMI) 20.54–25.76 kg/m^2^ were included in the study (S1 Table). After informed consent, blood samples (40 ml) were collected either under fasting conditions or 20 minutes after a high caloric (800 kcal) breakfast containing 21.96 g protein, 106.56 g carbohydrate and 32.09 g lipids (15.43 g saturated fat, 12.88 g monounsaturated fat and 2.12 g polyunsaturated fat) (PP state). Blood was centrifuged at 1700×*g* for 15 minutes at 4°C. Serum was collected, diluted 1:2 in filtered phosphate buffered saline (PBS) and centrifuged again at 2,000×*g* at 4°C for 30 minute to clear any remaining cells.

### 2.3 Exosome isolation from serum

Exosomes were obtained from serum samples as described [13]. Briefly, serum was centrifuged at 12,000×*g* for 60 minutes. Supernatants were then ultracentrifuged at 110,000×*g* for 2 hours. Pellets were diluted in PBS, filtered through a 0.22 μm filter and ultracentrifuged again at 110,000×*g* for 70 minutes. Pellets were washed with PBS by ultracentrifugation at 110,000 g for 70 min. For Western blot analysis pellets were suspended in RIPA (Sigma, R0278). For nanoparticle tracking analysis (NanoSight Ltd., Minton Park, Amesbury, UK), electron microscopy, labelling and functional assays, exosomes were suspended in PBS. All centrifugation steps were performed at 4 °C.

### 2.4 Electron microscopy

Electron microscopy was performed as described [13]. Briefly, exosome pellets obtained from serum were suspended in 100 μl of PBS, loaded onto Formwar carbon-coated grids and contrasted with 2% uranyl acetate. For immunogold staining the grids were placed into a blocking buffer step for 1 hr. Without rinsing, the grids were immediately placed into the primary antibody at the appropriate dilution overnight at 4°C (monoclonal anti-human-CD36 1:10, clone 99.1, bioceros; monoclonal anti-CD63 1:10, Abcam). As controls, some grids were not exposed to the primary antibody. The next day the grids were rinsed with PBS then floated on drops of IgA protein attached with 10nm gold particles (AURION, Hatfield, PA) for 1 hour at room temperature. Grids were rinsed with PBS and were placed in 1% Glutaraldehyde in 0.1M Phosphate buffer for 15 minutes. After rinsing in PBS and distilled water the grids were contrasted and embedded in a mixture of 2% methyl cellulose and 1% uranyl acetate. The grids were examined with a Tecnai G2 Spirit transmission electron microscope (TEM) (FEI Europe, Eindhoven, The Netherlands) and images were recorded using a Morada CCD camera (Olympus Soft Image Solutions GmbH, Münster, Germany).

### 2.5 Western blotting

Exosomes were lysed in RIPA buffer (1% NP40, 0.5% deoxycholate, 0.1% sodium dodecyl sulphate in Tris-buffered saline; Sigma, R0278) with complete protease inhibitors (Roche Diagnostics). Protein concentration was determined using the Qubit Protein Assay Kit (Invitrogen). Proteins were separated on 10% SDS-polyacrylamide gels and transferred to polyvinylidene difluoride (PVDF) membranes. Antibodies used were anti-CD36, anti-CD63, anti-CD9, anti-CD81 (Abcam, Cambridge UK). Detection was carried out with peroxidase-conjugated secondary antibodies using the ECL Plus Reagent (Amersham, GE Healthcare).

### 2.6 Exosome capture beads for FACS analysis

First, streptavidin-dynabeads of 10 μm of diameter (Thermo Fisher Scientific) were incubated with CD81 and CD9 biotinylated antibodies. Then, isolated exosomes from F/PP states were quantified by BCA and 30 μg/mL were incubated o.n with CD81 and CD9 coated dynabeads (Exosome capture beads). To evidence the presence of CD63 and CD36 in the circulating exosomes, exosome capture beads (loaded with exosomes) were incubated 1/100 in PBS during 1 h at R.T with CD63-ALX647 (BD Biosciences) and CD36-V450 (BD Biosciences) antibodies. After PBS washing exosome capture beads were analyzed by FACS. To analyze exosome BODIPY-FFA uptake by FACS, circulating exosomes were assay for BODIPY-FFA uptake (as in section 2.9) and then, 30 μg/mL of these exosomes were incubated o.n. with the coated dynabeads (Exosome capture beads). After PBS washing, dynabeads were analysed by FACS.

### 2.7 Cell culture

Primary human cardiac microvascular enothelial cells (hCMVECs, Promocell), were grown in EBM-2 BulletKit medium (Lonza, Basel, Switzerland). For primary neonatal rat cardiomyocytes cultures, one- to 2-day-old rat pups were sacrificed, hearts were excised, auricles were removed, and ventricles were minced. Primary cardiac cells (CM) were isolated using the Worthington Neonatal Cardiomyocyte Isolation System (Worthington Biochemical Corporation, Freehold, NJ, USA). CMs were cultured in Complete DMEM-high glucose, with L-glutamine, sodium pyruvate, 10% FBS, and 1% penicillin–streptomycin. All cells were cultured in a humidified incubator at 37°C and 5% CO_2_.

### 2.8 Lipid content assay in circulating exosomes by flow cytometry

Lipid content in exosomes was determined by flow cytometry quantification using Nile Red staining of neutral and amphipathic lipids in F/PP exosomes. Exosomes were incubated with Nile Red at a final concentration of 10 μg/ml at 37 °C for 30 minutes. Samples were then washed with PBS and suspended in 120 μl of PBS for flow cytometry analysis in FL2 (neutral lipids) and FL3 channels (amphipathic lipids).

### 2.9 *In vitro* BODIPY uptake by circulating exosomes

Isolated exosomes from F/PP were incubated with the fluorescent fatty acid analogue BODIPY 500/510 at 1.6 μg/ml for 10 minutes at 37°C. After washing with PBS and ultracentrifugation (as above) to re-isolate the exosomes, the fluorescence intensity of samples was measured with flow cytometry. To assess CD36-mediated FFA uptake, circulating exosomes from F and PP states were incubated at 4°C overnight with or without an anti-CD36 antibody (clone 99.1, Bioceros). After washing, an *in vitro* BODIPY uptake assay was performed as described above to assess the contribution of CD36 to FFA uptake.

### 2.10 Exosomal uptake of lipids from patient serum

Circulating exosomes were harvested as above from fasting or postprandial states. From the same subjects, an equal volume of serum was taken, which was then diluted 1:2 with PBS, followed by ultracentrifugation at 110,000×*g* overnight to deplete the sample of exosomes [13]. Previously collected exosomes were then incubated with exosome-depleted serum for 10 minutes at 37°C to determine their ability to take-up lipids from the depleted serum. After washing with PBS and ultracentrifugation, total lipid content in circulating exosomes was determined by flow cytometry analysis of Nile Red staining as described in section 2.8.

### 2.11 *In vitro* delivery of BODIPY in circulating exosomes to cardiac cells

F and PP circulating exosomes were isolated and loaded with BODIPY as described in section 2.9. Exosomes were quantified for their protein content with the BCA Protein Assay Kit (Pierce, Thermo Scientific). Equal amounts of exosomes were added to cardiac cell cultures (hCMVEC and primary rat neonatal cardiomyocytes) at a concentration of 20 μg/ml. After 10 minutes of incubation at 37°C cells, were washed with PBS and BODIPY fluorescence was analyzed by confocal microscopy and by flow cytometry for primary rat neonatal cardiomyocytes.

### 2.12 *In vivo* exosome delivery of BODIPY-FFA to the heart

F and PP circulating exosomes were loaded with Bodipy as described in section 2.9. Exosomes were quantified for their protein content with the BCA Protein Assay Kit (Pierce, Thermo Scientific) and 100 μg/ml of BODIPY-FFA loaded exosomes were intravenously injected into the tail vein of NOD/SCID mice. After 1hour and a half, animals were sacrified, hearts were excised and BODIPY-FFA fluorescence was measured directly in the fresh organ with the IVIS Spectrum (PerkinElmer; λexcitation: 500; λemission: 530). Alternatively, we also marked the exosomes membranes with BODIPY TR Ceramide, but we did not observe significant differences in the IVIS (not Shown).

### 2.13 Statistics

Data are expressed as mean±SE. Comparisons between experimental conditions were performed with Students paired t-test or analysis of variance (ANOVA) for multiple comparisons. Analyses were conducted with GraphPad Prism 5 software (GraphPad Software Inc., La Jolla, CA). Differences were considered statistically significant at P<0.05 with a 95% confidence interval.

## 3. Results

**Fig 1:**
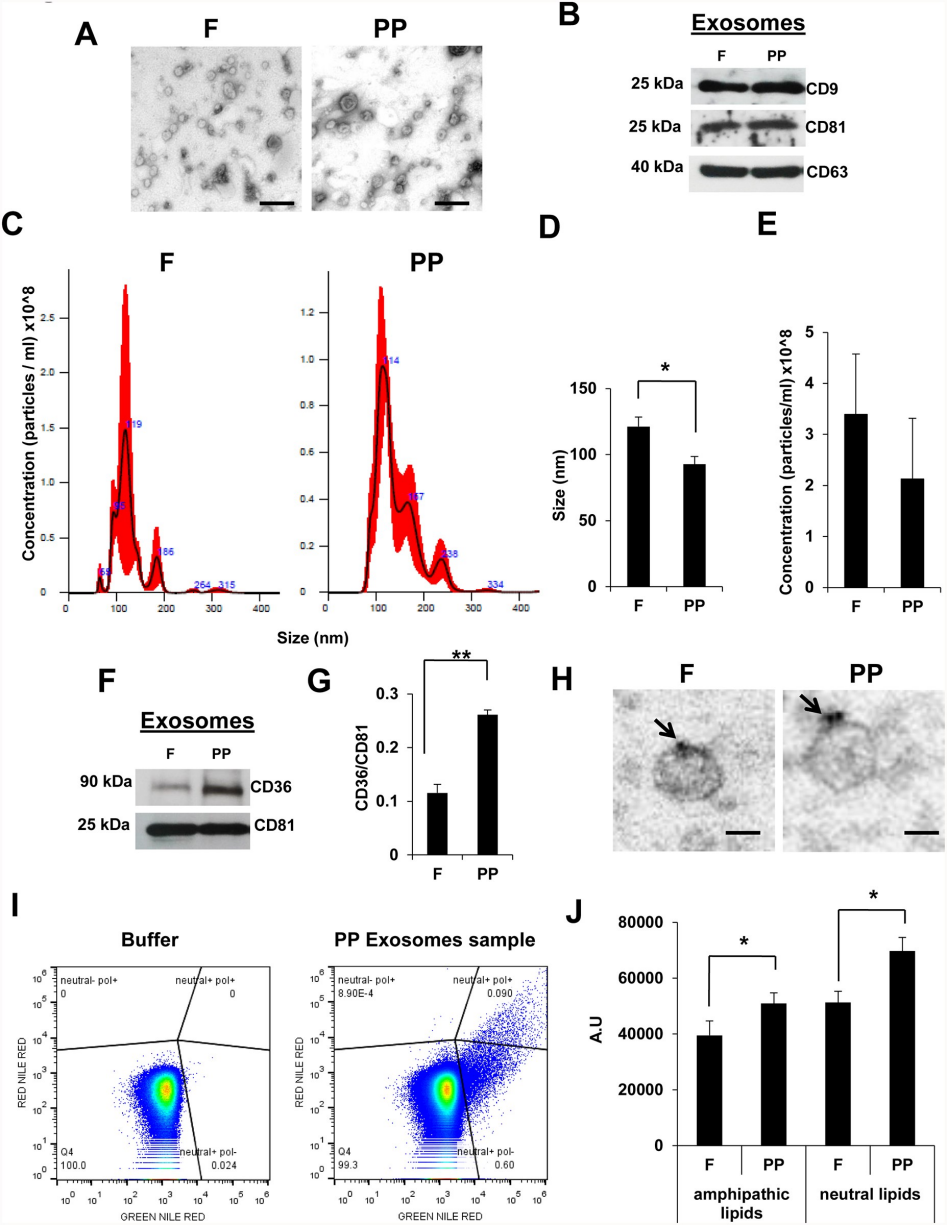
Circulating exosomes express CD36 and increase their lipid content after fat meal ingestion. **(A)** Representative electron microscopy images of isolated exosomes collected from serum of healthy donors after overnight fasting (F) and postpandrial (PP), 20 minutes after a high fat breakfast. Scale bars, 100 nm. **(B)** Representative western blot of tetraspanin expression in exosomes obtained from serum as in (A). **(C)** Representative images of exosomes analysed with a NanoSight NS300 instrument: particles/ml on the vertical axis and size in nanometers (nm) on the horizontal axis. The red shadow along the line shows the dispersion of the data obtained for that sample between the 3 videos of 1 minute each one. **(D)** The average size of F/PP circulating exosomes in serum analyzed by nanosight (N = 4, * *P*<0.05). **(E)** The average concentration of F/PP circulating exosomes in serum analyzed by nanosight (N = 4). **(F)** Western blotting of CD36 in exosomes isolated from F/PP serum. **(G)** Optical density quantification of western blot signals in (F) (N = 4 ** *P*<0.01). CD36 expression was normalized to the expression of CD81. (H) Anti-CD36 Immunogold electron microscopy images. The black arrows show the gold particles. Scale bars 50 nm. **(I)** Representative flow cytometry plots using Nile Red stain to quantify the lipid content in F/PP circulating exosomes. **(J)** Intensity signal of Nile Red stain analyzed by flow cytometry in F/PP circulating exosomes. (N = 4, * *P*<0.05)

Circulating exosomes isolated from fasting (F) and postprandial (PP) states were visualized by electron microscopy, revealing a typical bi-layered spheroidal shape and size (Fig 1A). Also western blotting of extracts detected equal levels of the common tetraspanin markers CD9, CD81 and CD63 (Fig 1B). Anti-CD63 Immunogold electron microscopy images are shown in S1 Fig. Counting and sizing of exosomes on the NanoSight instrument revealed a different size distribution between F and PP states (Fig 1C and 1D), with larger exosomes under the F state. No differences were found in the quantity of exosomes between the two states (Fig 1E).

Because CD36 is involved in FFA uptake in human tissues and it is known that circulatory CD36 in microparticles is related to metabolic pathology [14], we studied the possibility that CD36 was present in circulatory exosomes. Western blotting revealed CD36 expression in circulating exosomes from both fasting and postprandial states, although significantly higher levels of CD36 were found in exosomes isolated under the latter condition (Fig 1F and 1G). To corroborate CD36 presence at exosome surface we performed anti-CD36 Immunogold electron microscopy (Fig 1H). Moreover, to study CD36 in circulating exosomes by FACS, we used exosome capture dynabeads (which capture positive CD81 and CD9 exosomes) and we were able to detect CD36 signal in exosomes coated dynabeads as we shown in S1 Fig. Interestingly, PP exosomes samples showed highest levels of CD36 that F samples. We also studied the lipid composition of F and PP circulating exosomes using flow cytometry and Nile Red staining, which can discriminate between neutral and amphipathic lipids *via* the quantitative ratio of red and yellow emission [15, 16]. We found that levels of both amphipathic and neutral lipids were higher in postprandial-derived circulating exosomes than in equivalent fasting-derived exosomes (Fig 1I and 1J), suggesting a potential role for circulating exosomes in lipid metabolism after a high fat meal.

**Fig 2:**
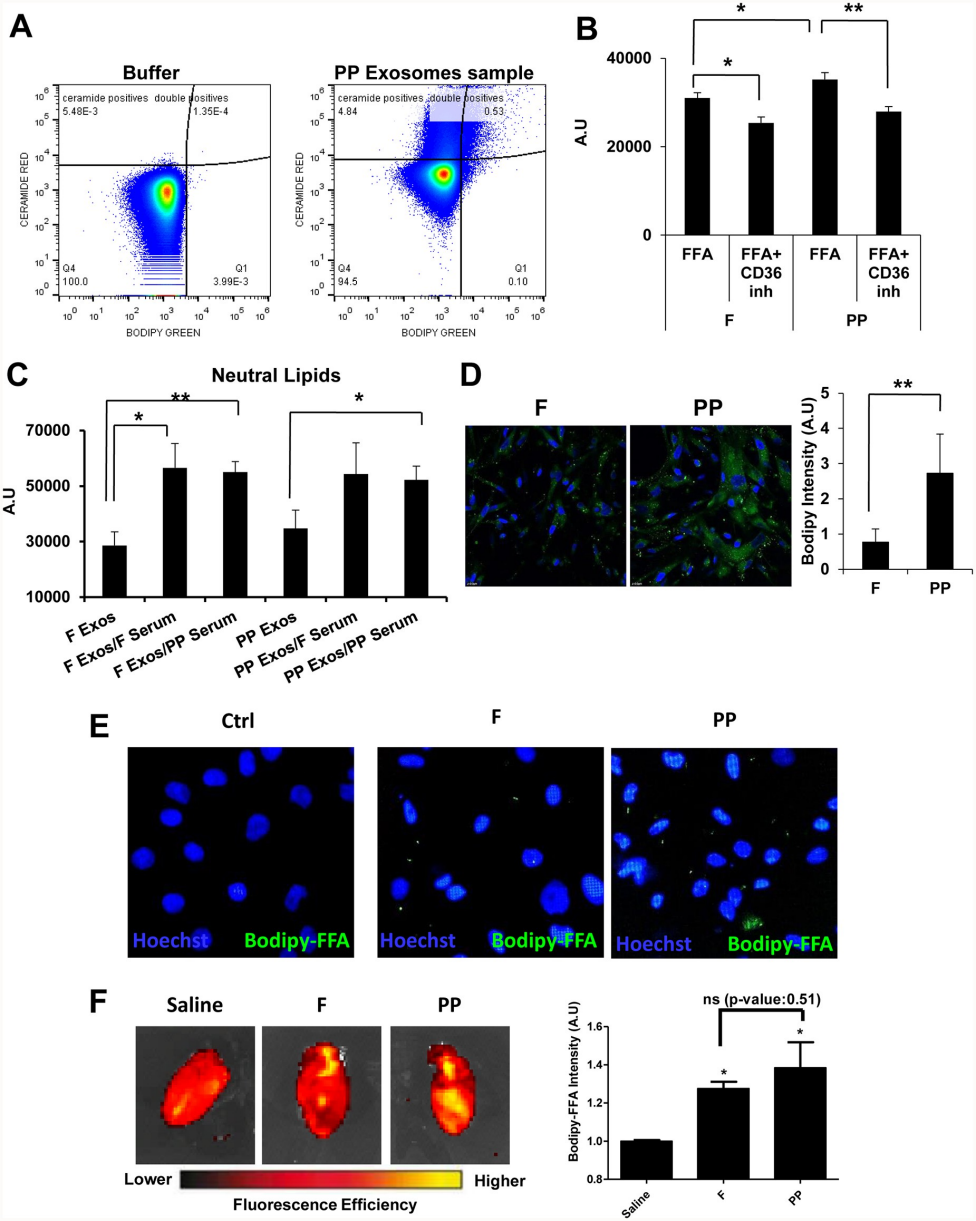
Circulating exosomes can incorporate and deliver FFAs into target cells. **(A)** Representative images of flow cytometry plots. Fasting (F)- and postpandrial (PP)-derived circulating exosomes were assayed *in vitro* for uptake of the green fluorescent fatty acid analogue BODIPY. **(B)** Graph shows the intensity signal of BODIPY in F/PP-derived circulating exosomes. Prior to the uptake assay, exosomes were treated or not with a CD36 antibody (+ CD36 Inh) (N = 5, * *P*<0.05, ** *P*<0.01). **(C)** F/PP-derived circulating exosomes were assayed to determine their lipid uptake from serum. F Exos: F circulating exosomes as control. F Exos/ F serum: F circulating exosomes after 10 minutes incubation with F exosome-depleted serum. F Exos/ PP serum: F circulating exosomes after 10 minutes incubation with PP exosome-depleted serum. PP Exos: PP circulating exosomes as control. PP Exos/ F serum: PP circulating exosomes after 10 minutes incubation with F exosome-depleted serum. PP Exos/ PP serum: PP circulating exosomes after 10 minutes incubation with PP exosome-depleted serum. Lipid content was determinated by Nile red staining and flow cytometry (N = 4, * *P*<0.05, ** *P*<0.01). **(D)** Representative images of living primary human cardiac microvascular enothelial cells (hCMVECs) treated during 10 minutes with F/PP-derived exosomes previously loaded with BODIPY (green fluorescence). Nuclei were stained with DAPI (blue). Graph shows the quantification of the green fluorescence (from BODIPY) in hCMVEC. (N = 4, ** *P*<0.01). **(E)** Representative image of living primary cardiac cells (CM; nucleus: blue) treated with F/PP circulating exosomes previously loaded with BODIPY-FFA (green fluorescence). Nucleus stain with Hoechst (blue) **(F)** F/PP exosomes previously loaded with BODIPY-FFA were intravenously injected into nod/scid mice. After 1.5 hour mice were euthanized and heart were collected. BODIPY-FFA fluorescence in the heart was detected using IVIS Spectrum (PerkinElmer; Red: less fluorescent signal; Yellow: more fluorescent signal). Graph showing BODIPY-FFA fluorescence (Ex: 500/ Em: 535) in the heart normalized vs autofluorescence detected in untreated hearts (Saline) (N = 3, * *P*<0.05).

As CD36 was present in circulating exosomes, we assessed the *ex vivo* ability of these exosomes to take-up the fluorescent BODIPY fatty acid analogue (BODIPY-FFA) (Fig 2A and 2B). We observed that whereas circulating exosomes harvested under either condition could incorporate BODIPY-FFA, greater amounts were incorporated from exosomes harvested under PP conditions (Fig 2A and 2B). To test whether the uptake of BODIPY-FFA was mediated by CD36, we pre-treated exosomal preparations with a CD36 blocking antibody, finding that BODIPY-FFA uptake was reduced in both cases, F and PP. Similar approach was tested using exosome capture beads. Results are shown in S2 Fig. These results suggest that FFA uptake in circulating exosomes is mediated, at least in part, by CD36.

A considerable degree of FFA spillover into the systemic circulation occurs under non-pathological conditions in the postprandial state [7]. Given our observation that circulating exosomes could incorporate FFAs *ex vivo*, we studied the possibility that circulating exosomes could takep-up lipids directly from serum. To do this, we isolated circulating exosomes from F and PP states and then incubated them for 10 minutes with serum from either of the two conditions (F or PP) that had been depleted of exosomes. We then measured the lipid composition of the exosomes by flow cytometry. We observed that exosomes from both conditions were able to incorporate neutral lipids by direct uptake from serum (Fig 2C). By contrast, no changes were observed for amphipathic lipids (data not show). These results strongly suggest that exosomal FFA uptake can take place directly in the bloodstream.

Cardiac tissue is a major FFA consuming organ. Given our results showing that circulating exosomes can incorporate circulatory FFAs *in vitro*, we next investigated the capacity of circulating exosomes to be internalized into cardiac cells (both endothelial cells and structural cardiomyocytes) and deliver the BODPIY-FFA analogue. Since exosomes need to traverse the cardiac endothelial microvasculature before reaching the myocardium, we added circulating exosomes previously loaded with BODIPY-FFA to primary hCMVEC (Fig 2D) and to primary neonatal rat cardiomyocyte (Fig 2E). After 10 minutes incubation of cell cultures with equal amounts circulating exosomes isolated from F or PP states and loaded with BODIPY-FFA. We observed that incorporation of BODIPY-FFA was greater when hCMVEC was treated with PP-derived exosomes. Also, we observed BODIPY-FFA incorporation via exosomes in primary cardiomyocytes, but not significant difference was observed when we analysed it by FACS (S3 Fig). These results thus suggest that circulating exosomes can deliver FFAs to cardiac cells *in vitro*. Finally, to evaluate the ability of circulating exosomes to transfer FFA to the heart *in vivo*, F and PP exosomes previously loaded with BODIPY-FFA were injected intravenously in NOD/SCID mice. After 1 hour and a half, hearts were collected and BODIPY-FFA fluorescence was measured using IVIS Spectrum (PerkinElmer). Hearts from mice previously injected with BODIPY-FFA loaded exosomes showed higher fluorescent intensity in the Bodipy-FL emission window although we could not detect differences in fluorescence intensity between F and PP exosomes probably due to the sensitivity of the technique (Fig 2F). These results suggest that circulating exosomes are able to deliver FFA from the bloodstream to the heart *in vivo*.

## 4. Discussion

Circulating FFAs are crucial as energy substrates and for the synthesis of most lipids. Nevertheless, oversupply of FFAs disrupts cellular acid-base homeostasis, alters the integrity of cellular membranes and elicits the generation of harmful bioactive lipids [17]. Thus, the regulation of circulating FFA is fundamental to the physiology of tissues. In the fasting state, the principal source of FFAs to most tissues originates from AT lipolysis. After a meal, fats are carried by chylomicrons to the bloodstream for final storage in the AT and this process generates FFA spillover, which contributes to the circulating FFA pool [7]. Our results allow us to propose a role for circulating exosomes in FFA delivery from the bloodstream to tissues.

We demonstrate that circulating exosomes express functional CD36. Moreover, we found that the amount of CD36 in exosomes increases in the postprandial state, prompting us to compare the lipid composition of exosomes in the F and PP states. We found that PP circulating exosomes have a higher lipid content than those in the F state. This result suggests two possibilities: the first is that after a high caloric meal, exosomes are released into the bloodstream with a higher load of lipids; alternatively, circulating exosomes support CD36-mediated lipid uptake from the bloodstream, especially in the postprandial state, where circulating exosomes showed higher levels of CD36. We studied the second scenario, but further studies will be necessary to examine the first scenario and to elucidate the origin of the circulating exosomes. We show that circulating exosomes can increase their lipid content by direct incubation with the serum, which supports the idea of bloodstream FFA uptake by circulating exosomes. Moreover, we observed that circulating exosomes could incorporate *in vitro* the florescent FFA analogue BODIPY in a CD36-dependent manner. In a cardiac context, a study by Tanaka and co-workers showed that patients carried mutations in CD36 were unable to uptake a labelled FFA analogue by the cardiac tissue [18]. Moreover, expression of CD36 has been linked to cardiac hypertrophy, tolerance to ischaemia and diastolic dysfunction in rodent models [19]. Interestingly, CD36 has been implicated in pathological conditions associated with metabolic dysregulation, including insulin resistance, obesity, diabetes and atherosclerosis [20–22]

The dysregulation of the mechanisms that control the circulating FFA levels are strongly related with metabolic pathologies but exist controversy to find a clear causal-effect relation between the circulating FFA level and the metabolic pathology [23]. The most accepted theory tells that dysregulation of adipose tissue lipolysis results in increased circulating FFA levels, which contribute to develop insulin resistance and type 2 diabetes mellitus. However, some authors shown evidence supporting the idea that this relation needs to be revised [7]. Here, we show data supporting a new circulating FFA delivery mechanism.

Finally, BODIPY-FFA-loaded exosomes could be internalized by cardiac endothelial cells and cardiomyocytes, suggesting an exosome delivery mechanism of FFA into cardiac tissue. Moreover, our *in vivo* data show that circulating exosomes that previously uptake BODIPY-FFA *ex vivo*, are able to bring it to the heart from the mice circulation. Thus, we propose a novel mechanism whereby circulating exosomes are able to incorporate and deliver FFAs from the bloodstream into tissues. Further studies are needed to examine in detail how this “exosomal FFA delivery” system might work, and in particular its potential involvement in patients with metabolic pathologies such as obesity, diabetes and atherosclerosis.

## Supporting information

S1 Fig **(A)** Representative immunogold electron microscopy (anti-CD63) images of isolated exosomes collected from serum of healthy donors after overnight fasting (F) and postpandrial (PP), 20 minutes after a high fat breakfast. Scale bars, 80 nm. **(B)** Representative FACS of exosome capture dynabeads incubated with anti-CD36-V450 or anti-CD63-Alexa647 alone as a control (upper panel) or previously coupled with circulating exosomes. “X” axis: CD36-V450 or CD63-Alexa647 signal; “Y” axis: SSC-A. **(C)** Quantification of the FACS percentage of exosome capture beads (E.C. Beads) incubated with exosomes obtained from F and PP serum as in (A), positive for CD63-Alexa 647 or CD36-V450 signal (N = 2, * P<0.*05)*.
(TIF)

S2 Fig **(A)** Representative image of FACS with exosome capture beads (E. C. Beads). F/PP circulating exosomes were assay to uptake *in vitro* the green fluorescent FA analogue bodipy. Previous to the uptake assay exosomes were treated or not with a CD36 inhibitor (+ CD36 Inh) **(B)** Graphic shows the BODIPY-FFA loaded exosome captured in the exosome capture beads from F/PP circulating exosomes previously incubated or not with a CD36 inhibitor (+ CD36 Inh). Samples were normalized to F exosomes (N = 3, * *P*<0.05, ** *P*<0.01).
(TIF)

S3 Fig **(A**) Representative FACS histograms of primary cardiac cells (CM) treated with F- or PP circulating exosomes previously loaded with BODIPY-FFA. **(B)** Flow cytometry quantification of green fluorescence resulting from incorporation of BODIPY-FFA-loaded exosomes (N = 3, *P<0.05) in primary cardiac cells. AU stands for arbitrary units of fluorescence in all panels. Arbitrary units were normalized to control cells values.
(TIF)

S1 Table Blood donors data. (XLSX)

## Abbreviations

AT: adipose tissue
BMI: body mass index
CM: cardiomyocyte
F: fasting
FFA: free fatty acid
hCMVEC: human cardiac microvascular endothelial cells
LPL: lipoprotein lipase
PP: postprandial
TG: triglyceride
