# Supplementary figures and images for: Circulating exosomes deliver free fatty acids from the bloodstream to cardiac cells: Possible role of CD36
Source: PLoS One. 2019 May 29;14(5):e0217546. doi: 10.1371/journal.pone.0217546 (PMC6541372; doi:10.1371/journal.pone.0217546)

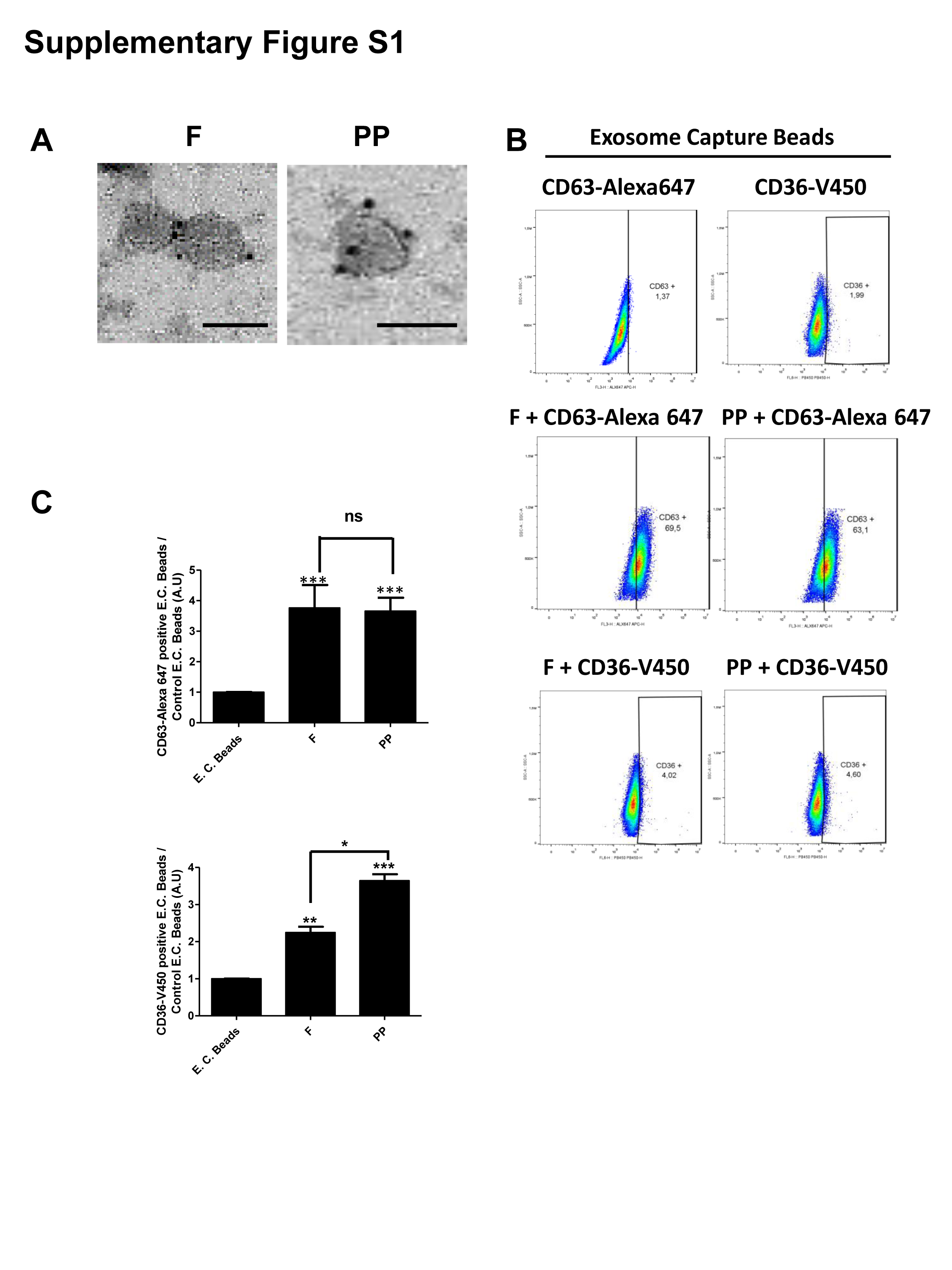

Supplement: S1 Fig — (A) Representative immunogold electron microscopy (anti-CD63) images of isolated exosomes collected from serum of healthy donors after overnight fasting (F) and postpandrial (PP), 20 minutes after a high fat breakfast. Scale bars, 80 nm. (B) Representative FACS of exosome capture dynabeads incubated with anti-CD36-V450 or anti-CD63-Alexa647 alone as a control (upper panel) or previously coupled with circulating exosomes. “X” axis: CD36-V450 or CD63-Alexa647 signal; “Y” axis: SSC-A. (C) Quantification of the FACS percentage of exosome capture beads (E.C. Beads) incubated with exosomes obtained from F and PP serum as in (A), positive for CD63-Alexa 647 or CD36-V450 signal (N = 2, * P<0.05). (TIF) [file pone.0217546.s001.tif]

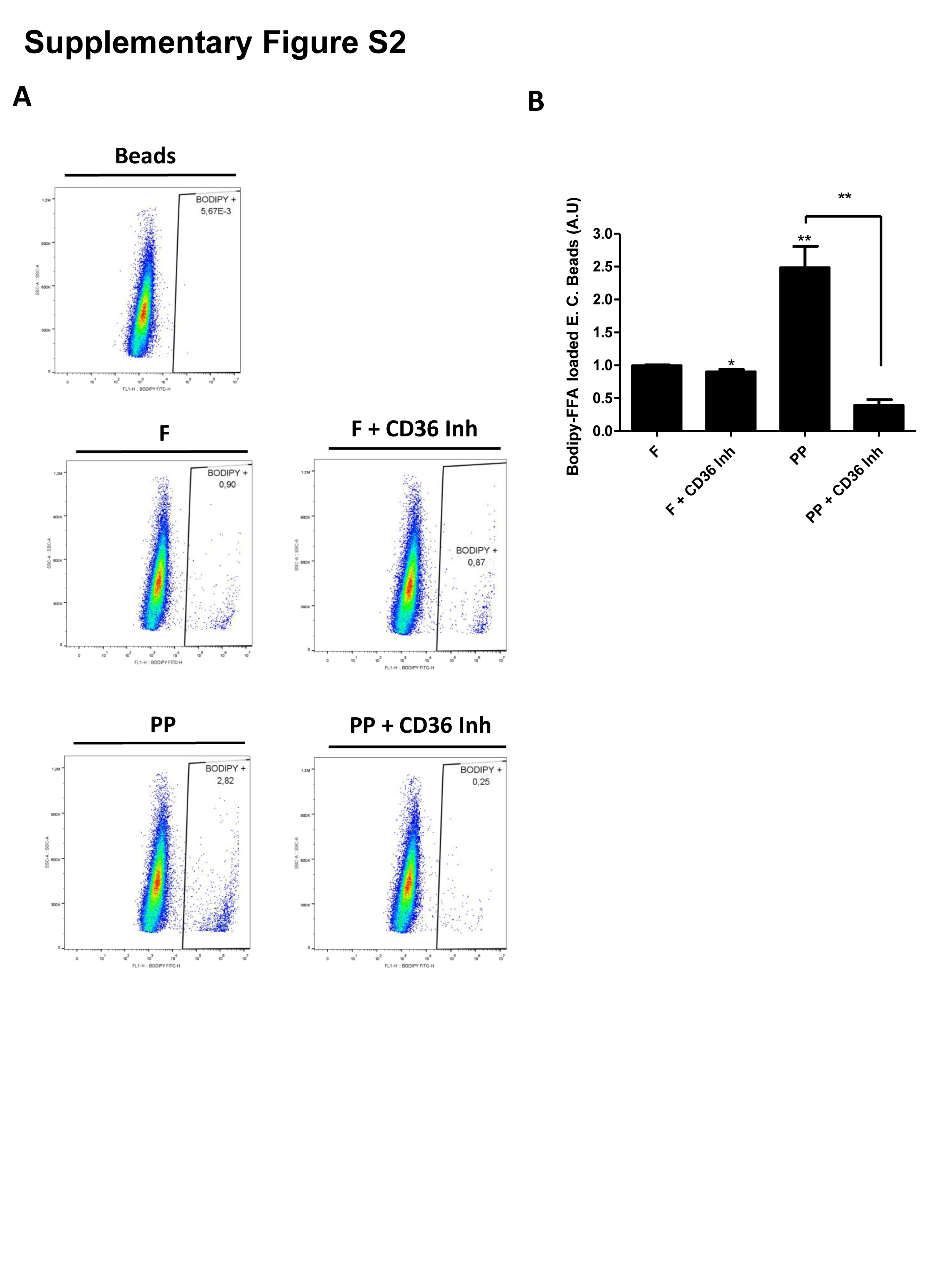

Supplement: S2 Fig — (A) Representative image of FACS with exosome capture beads (E. C. Beads). F/PP circulating exosomes were assay to uptake in vitro the green fluorescent FA analogue bodipy. Previous to the uptake assay exosomes were treated or not with a CD36 inhibitor (+ CD36 Inh) (B) Graphic shows the BODIPY-FFA loaded exosome captured in the exosome capture beads from F/PP circulating exosomes previously incubated or not with a CD36 inhibitor (+ CD36 Inh). Samples were normalized to F exosomes (N = 3, * P<0.05, ** P<0.01). (TIF) [file pone.0217546.s002.tif]

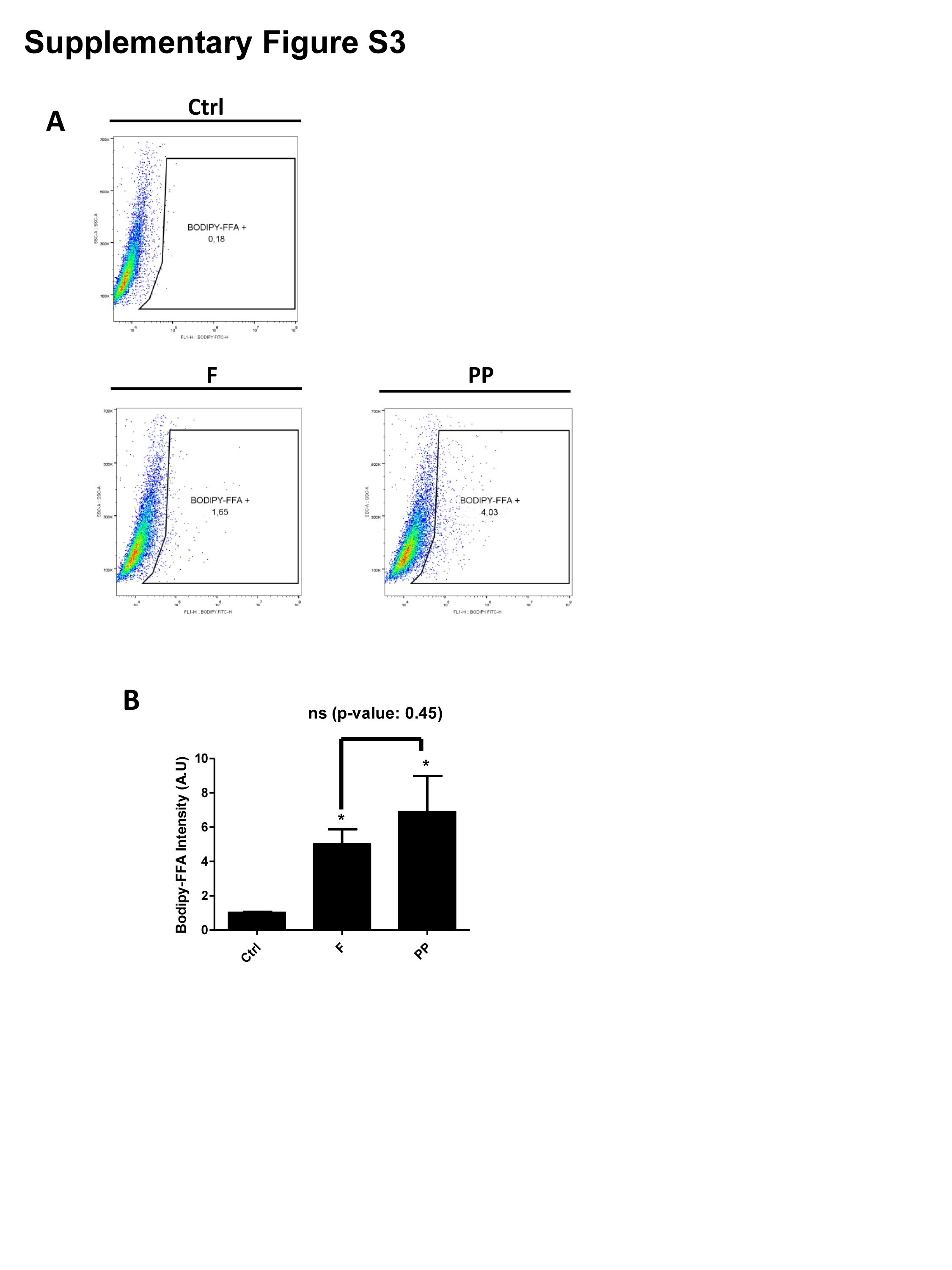

Supplement: S3 Fig — (A) Representative FACS histograms of primary cardiac cells (CM) treated with F- or PP circulating exosomes previously loaded with BODIPY-FFA. (B) Flow cytometry quantification of green fluorescence resulting from incorporation of BODIPY-FFA-loaded exosomes (N = 3, *P<0.05) in primary cardiac cells. AU stands for arbitrary units of fluorescence in all panels. Arbitrary units were normalized to control cells values. (TIF) [file pone.0217546.s003.tif]
